# Causal role of gut microbiota, serum metabolites, immunophenotypes in myocarditis: a mendelian randomization study

**DOI:** 10.3389/fgene.2024.1382502

**Published:** 2024-08-30

**Authors:** Kaiyuan Li, Peng Liu, Xiuqi Wang, Zhipeng Zheng, Miao Liu, Jun Ye, Li Zhu

**Affiliations:** ^1^ Graduate School of Dalian Medical University, Dalian Medical University, Dalian, China; ^2^ Department of Cardiovascular Medicine, The Affiliated Taizhou People’s Hospital of Nanjing Medical University, Taizhou, China; ^3^ Department of Cardiovascular Medicine, The Second Affiliated Hospital of Nanchang University, Nanchang, China; ^4^ Department of Cardiovascular Medicine, Center Hospital of Shandong First Medical University, Jinan, China

**Keywords:** gut microbiota, serum metabolites, immunophenotypes, myocarditis, mendelian randomization study

## Abstract

**Background:**

The intricate relationship among gut microbiota, serum metabolites, and immunophenotypes may significantly impact myocarditis. However, direct causal links between these domains and myocarditis are not well understood.

**Methods:**

The study performed Mendelian randomization (MR) analysis using genetic data from public sources. Exposure data included 211 gut microbiota, 486 serum metabolites, and 731 immunophenotypes from Mibiogen, the Metabolomics GWAS server, and GWAS catalog databases. Single nucleotide polymorphisms (SNPs) were selected as instrumental variables based on established criteria. Myocarditis data from GWAS (427,911 participants, 24, 180, 570 SNPs) were used as the outcome variable. MR analysis was conducted using Inverse Variance Weighting (IVW), with Cochran’s Q test for heterogeneity and Egger’s intercept to assess horizontal pleiotropy.

**Results:**

9 gut microbiota, 10 serum metabolites, and 2 immunophenotypes were negatively associated with myocarditis risk. In contrast, 5 gut microbiota, 12 serum metabolites, and 7 immunophenotypes were positively associated with myocarditis risk (all, *P* < 0.05). Sensitivity analyses confirmed the stability of these results.

**Conclusion:**

This MR study suggests that gut microbiota, serum metabolites, and immunophenotypes may causally influence myocarditis risk. These findings provide genetic evidence for myocarditis etiology and could inform future precision prevention and treatment strategies.

## 1 Introduction

Myocarditis is characterized by inflammatory infiltration of the myocardium, predominantly caused by viral infections, autoimmune responses, bacterial infections, or other inflammatory agents. Increasingly, research suggests a significant association between myocarditis and the gut microbiota, serum metabolites, and the immune system. Although less common than other heart diseases, myocarditis can persist for extended periods in certain populations. It can potentially progress to a chronic stage, resulting in myocardial tissue fibrosis, hypertrophy, and myocyte apoptosis. This progression can ultimately lead to life-threatening conditions such as circulatory failure and lethal ventricular arrhythmias (Sagar et al., 2012; Vdovenko and Eriksson, 2018).

The heart contains a diverse array of immunophenotypes crucial for maintaining tissue integrity. These immunophenotypes play a pivotal role in cardiovascular health and disease (Wernersson and Pejler, 2014). Current research has shown that their role extends beyond host defense. Immunophenotypes are now known to be involved in development, tissue dynamic homeostasis, and repair (Saparov et al., 2017). For example, cardiac fibroblasts and endothelial cells have immune functions essential for maintaining and restoring homeostasis without infection (Drummer et al., 2021; Rose, 1998). However, there have been no systematic large population studies on immunophenotypes associated with the pathogenesis of myocarditis until now.

The human gut microbiota is a complex and dynamic group of microorganisms present in the gastrointestinal tract, which is slowly becoming a hot topic in human pathogenesis and treatment (Adak and Khan, 2019; Xie et al., 2024). The intricate symbiosis between the gut microbiota and its host has profound implications. It influences metabolic functions, immune system development, and even behavioral aspects through the gut-heart axis (Zhao et al., 2022). Serum metabolites, closely related to the gut microbiome, are produced through its metabolic activities. These metabolites affect the host’s metabolic, immune, and neurological functions (Sha et al., 2023). While gut microbiota and serum metabolites play important roles in acute coronary syndromes, studies on their involvement in myocarditis are still lacking (Qiu et al., 2023).

**FIGURE 1:**
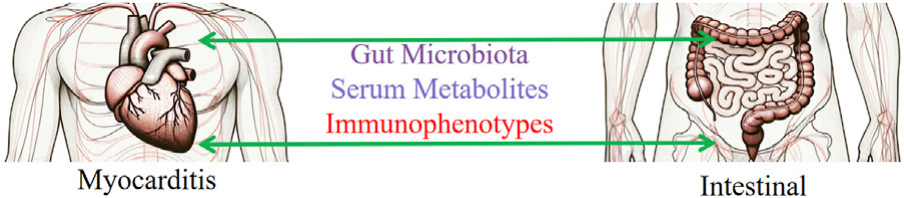
Digestive organs and heart interplay through gut microbiota, serum metabolites, and immunophenotypes.

Mendelian randomization (MR) studies leverage genome-wide association data (GWAS) and bioinformatics analyses to uncover causal relationships between exposures and outcomes (Wang et al., 2023). MR functions as a naturally occurring large-scale randomized controlled trial, adhering to fundamental MR principles (Birney, 2022; Davey Smith and Hemani, 2014). This study explored and determined the causal relationships between gut microbiota, serum metabolites, immunophenotypes, and myocarditis using MR analysis (Figure 1, Table 1).

**TABLE 1:** Details of the genome-wide association studies and datasets used in our analyses.

Exposure/Outcome	Year	Author	Participants	Number of SNPs	Web source if publicly
Myocarditis (ebi-a-GCST90018882	2021	Sakaue S	427,911 individuals (633 cases and 427,278 controls) of European ancestry	24,180,570	https://gwas.mrcieu.ac.uk/datasets/ebi-a-GCST90018882 (Access time:4 February 2024)-
211 gut microbiota	2021	Kurilshikov	18,340 individuals of Asian and European ancestry	NA	https://mibiogen.gcc.rug.nl (Access time:4 February 2024)
486 serum metabolites	2014	Shin et al	7,824 individuals of European ancestry	approximately 2.1 million SNPs	(http://metabolomics.helmholtz-muenchen.de/gwas/(Access time:4 February 2024)
731 immunophenotypes	2020	Orrù V, et al	3,757 individuals of European ancestry	Approximately 22 million SNPs	https://www.ebi.ac.uk/gwas/downloads/summary-statistics (Access time:4 February 2024)

## 2 Materials and methods

### 2.1 Study design

**FIGURE 2:**
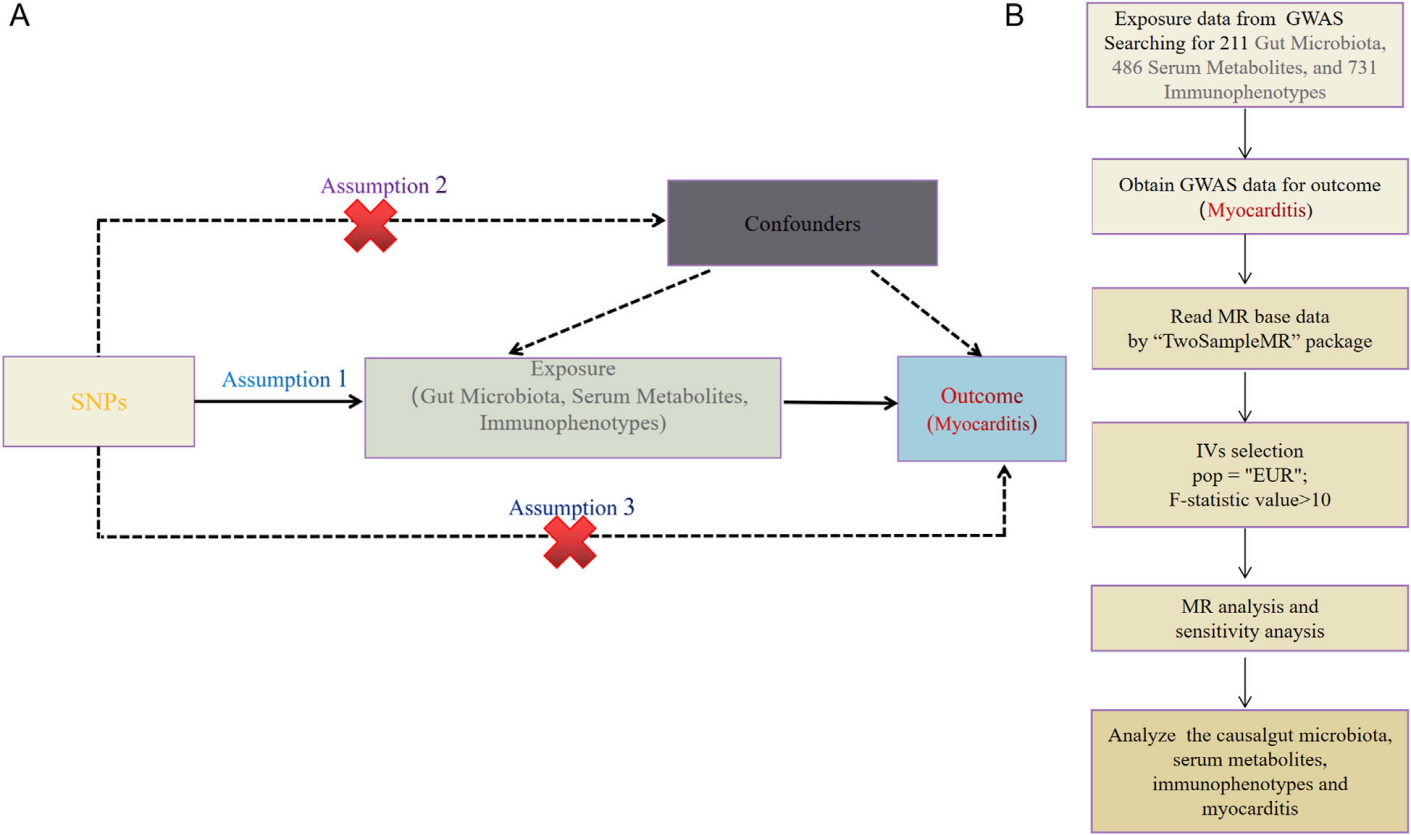
**(A)** This study strictly followed the three core assumptions of MR studies. **(B)** Workflow of the MR.

The causal relationship between the two samples was explored using 211 gut microbiota, 486 serum metabolites, and 731 immune cell characteristics as exposure factors, with myocarditis as the outcome. The study methods were compliant with the STROBE-MR checklist (Skrivankova et al., 2021). MR methods employ genetic variants as instrumental variables (IVs) to represent potential risk factors, enabling causal inferences from observational data: IVs in causal inference must satisfy three key assumptions. To ensure the validity of MR analyses, the selected IVs must satisfy three core assumptions: 1) genetic variation is directly related to exposure; 2) genetic variation is independent of possible confounders between exposure and outcome; 3) genetic variation does not affect outcome through pathways other than exposure (Figure 2A).

### 2.2 GWAS data sources and selection of IVs

Data on 211 gut microbiota traits were downloaded from the MiBioGen website (https://mibiogen.gcc.rug.nl/). The dataset included 16S rRNA gene sequencing profiles and genotyping data from 18,340 subjects across 11 countries. These data encompassed 211 traits within 35 families, 20 orders, 16 phyla, 9 orders, and 131 genera. Due to the limited availability of single nucleotide polymorphisms (SNPs) loci with *P* < 1 × 10^−8^ for gut microbiota, SNPs loci with *P* < 1 × 10^−5^ were selected. The loci obtained from the screening were used as instrumental variables in place of the clinical risk exposure factor gut microbiota. Data from SNPs with chained unbalanced aggregates were subsequently removed, with removal conditioned on LD (r^2^ < 0.001, distance <10, 000 kb), and an F-statistic >10 was used to exclude the effect of weak IVs bias (Yu et al., 2023).

Data on 486 serum metabolites were downloaded from GWAS data, involving 7,824 adult individuals from two European population studies. SNPs were screened based on *P* < 1 × 10^−5^, r^2^ < 0.01, distance <500 kb, and F > 10 to exclude the effect of weak IVs bias (Zou et al., 2024).

Data on 731 immunophenotypes were downloaded from the GWAS catalog (accession numbers GCST0001391 to GCST0002121). This dataset included 3,757 European individuals with a total of 731 immunophenotypes, including 118 absolute cell counts, 389 median fluorescence intensities, 32 morphological parameters, and 192 relative cell counts. The immunophenotypes as exposed IVs fulfilled the following conditions: genome-wide significant level (*P* < 1 × 10^−5^), removal of the linkage disequilibrium threshold (r^2^ < 0.001, distance <10,000 kb), and F > 10 to exclude the effect of weak IVs bias (Tang et al., 2024).

The studies included in our analysis were approved by the relevant institutional review boards, and participants provided signed informed consent.

### 2.3 Data source of myocarditis

Myocarditis data (ebi-a-GCST90018882) were obtained from the European Bioinformatics Institute (EBI), which enrolled a total of 427,911 European individuals (Ncase = 633, Ncontrol = 427,278) and contained 24, 180, 570 SNPs (Figure 2B).

### 2.4 MR analysis

The Inverse Variance Weighting (IVW) method was used as the primary estimation technique to assess the causal association between exposure and outcome. In this method, the causal effects of different genetic variants on a trait are weighted by inverse variance, making IVW the standard for MR analysis. A random effects model was applied if heterogeneity existed; otherwise, a fixed effects model was used. The results were expressed as odds ratios (OR) with 95% confidence intervals (CI) to estimate the causal effect of exposure on the outcome.

### 2.5 Statistical analysis

To ensure the stability and reliability of the results of the MR analyses, the Cochran Q test was used in this study to assess the heterogeneity of the SNPs, and the MR Egger intercept test was performed to detect the presence of horizontal pleiotropy. *P* < 0.05 was considered to be the presence of heterogeneity or horizontal pleiotropy.

## 3 Results

### 3.1 14 gut microbiotas may potentially influence the risk of developing myocarditis

**FIGURE 3:**
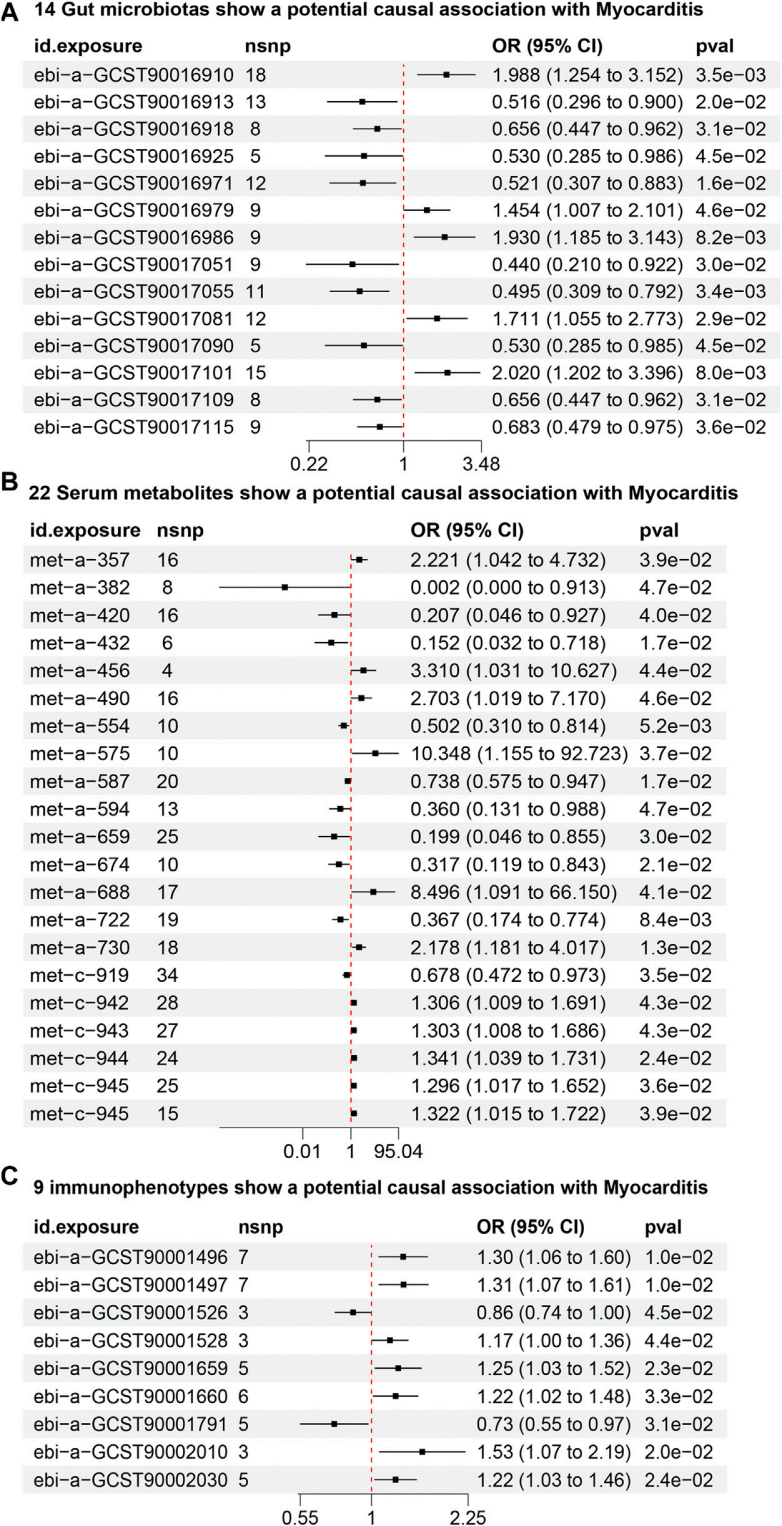
**(A)** 14 gut microbiotas may potentially influence the risk of developing myocarditis. **(B)** 22 serum metabolites may potentially influence the risk of developing myocarditis. **(C)** 9 Immunophenotypes may potentially influence the risk of developing myocarditis.

From the gut microbiota dataset, 178 SNPs were screened (Supplementary Table S1). IVW analysis identified 14 gut microbiotas as potentially causally associated with myocarditis (Figure 3A) (Supplementary Table S2). 5 gut microbiotas were positively associated with the risk of developing myocarditis: class Bacilli (id.1673; OR = 1.98, 95% CI = 1.25–3.15, *P* = 0.003), order Lactobacillales (id.1800; OR = 2.02, 95% CI = 1.21–3.38, *P* = 0.007), genus Defluviitaleaceae UCG011 (id.11287; OR = 1.93, 95% CI = 1.18–3.14, *P* = 0.008), an unknown genus (id.1868; OR = 1.71, 95% CI = 1.05–2.77, *P* = 0.029), and genus *Clostridium* innocuum group (id.14397; OR = 1.45, 95% CI = 1.00–2.10, *P* = 0.045). Nine gut microbiotas, including genus Bilophila (id.3170; OR = 0.52, 95% CI = 0.31–0.88, *P* = 0.015) and class Clostridia (id.1859; OR = 0.51, 95% CI = 0.29–0.89, *P* = 0.019), were negatively associated with the risk of developing myocarditis.

### 3.2 22 Serum metabolites may potentially influence the risk of developing myocarditis

From the dataset of serum metabolites, 361 SNPs were screened (Supplementary Table S3). IVW analysis found 22 serum metabolites to be potentially causally associated with myocarditis (Figure 3B) (Supplementary Table S2). 12 serum metabolites, including 10-heptadecenoate (OR = 50.77, 95% CI = 2.26–1136.45, *P* = 0.013) and valerate (OR = 10.34, 95% CI = 1.15–92.72, *P* = 0.036), were positively associated with the risk of myocarditis. Conversely, 10 serum metabolites, including N1-methyladenosine (OR = 0.01, 95% CI = 0.00–0.91, *P* = 0.046) and gamma-glutamylisoleucine (OR = 0.19, 95% CI = 0.04–0.85, *P* = 0.029), were negatively associated with the risk of myocarditis.

### 3.3 9 Immunophenotypes may potentially influence the risk of developing myocarditis

From the dataset of immunophenotypes, 51 SNPs were screened (Supplementary Table S4). IVW analysis identified 9 immunophenotypes as potentially causally associated with myocarditis (Figure 3C) (Supplementary Table S2). 7 immunophenotypes were positively associated with the risk of developing myocarditis. These included CD39^+^ secreting CD4 regulatory T cells (OR = 1.30, 95% CI = 1.06–1.59, *P* = 0.011), CD39^+^ CD4 regulatory T cells (OR = 1.31, 95% CI = 1.06–1.60, *P* = 0.011), HLA DR on monocytes (OR = 1.53, 95% CI = 1.06–2.19, *P* = 0.019), CD39^+^ CD4^+^ T cells (OR = 1.25, 95% CI = 1.03–1.51, *P* = 0.022), CD39 on CD39^+^ activated CD4 regulatory T cells (OR = 1.22, 95% CI = 1.02–1.45, *P* = 0.021), CD39^+^ CD4^+^ T cell absolute count (OR = 1.22, 95% CI = 1.01–1.47, *P* = 0.031), and D33dim HLA DR+ CD11b- (OR = 1.16, 95% CI = 1.00–1.35, *P* = 0.044). 2 immunophenotypes were negatively associated with the development of myocarditis: CD33dim HLA DR + CD11b (OR = 0.85, 95% CI = 0.73–0.99, *P* = 0.044) and CD25 on naive-mature B cells (OR = 0.73, 95% CI = 0.55–0.97, *P* = 0.031).

### 3.4 No heterogeneity or horizontal pleiotropy found

No heterogeneity was found for the 14 gut microbiotas and 22 serum metabolites and 9 immunophenotypes analyzed in this study, while at the same time no horizontal pleiotropy was found to exist (Tables 2–4).

**TABLE 2:** Sensitivity analyses of the causal effect of gut microbiota on myocarditis.

Genomics	Expoure	Test for directional horizontal pleiotropy			Cochran’s Q test	
		Egger intercept	SE	*P*-Value	Q	*P*-Value
gut microbiota	class Bacilli id.1673	0.061	0.048	0.224	12.1	0.740
gut microbiota	class Clostridia id.1859	0.013	0.053	0.811	7.80	0.731
gut microbiota	class Lentisphaeria id.2250	−0.13	0.099	0.237	1.17	0.979
gut microbiota	family Actinomycetaceae id.421	0.048	0.081	0.593	2.76	0.430
gut microbiota	genus Bilophila id.3170	−0.028	0.096	0.78	9.30	0.503
gut microbiota	genus Clostridium innocuum group id.14397	0.002	0.121	0.987	3.41	0.845
gut microbiota	genus Defluviitaleaceae UCG011 id.11287	0.065	0.091	0.495	3.60	0.824
gut microbiota	genus Ruminiclostridium9 id.11357	0.015	0.114	0.9	7.65	0.365
gut microbiota	genus Ruminococcaceae UCG004 id.11362	0.065	0.113	0.58	8.80	0.456
gut microbiota	order Actinomycetales id.420	0.049	0.081	0.589	2.74	0.433
gut microbiota	order Lactobacillales id.1800	0.066	0.05	0.211	12.7	0.468
gut microbiota	order Victivallales id.2254	−0.13	0.099	0.237	1.17	0.979
gut microbiota	phylum Lentisphaerae id.2238	−0.135	0.101	0.224	1.41	0.985
gut microbiota	unknown genus id.1868	−0.003	0.072	0.967	9.72	0.465

**TABLE 3:** Sensitivity analyses of the causal effect of serum metabolites on myocarditis.

Genomics	Expoure	Test for directional horizontal pleiotropy			Cochran’s Q test	
		Egger intercept	SE	*P*-Value	Q	*P*-Value
serum metabolites	Biliverdin	0.002	0.027	0.944	10.9	0.693
serum metabolites	X-03003	−0.026	0.155	0.871	2.66	0.850
serum metabolites	N1-methyladenosine	0.093	0.081	0.293	0.49	0.998
serum metabolites	2-hydroxybutyrate (AHB)	0.049	0.034	0.172	9.34	0.809
serum metabolites	Aspartylphenylalanine	0.02	0.076	0.808	4.30	0.367
serum metabolites	Pyroglutamylglycine	0.026	0.107	0.828	0.421	0.810
serum metabolites	X-11261	−0.045	0.039	0.265	6.16	0.962
serum metabolites	X-11852	−0.034	0.039	0.417	5.26	0.729
serum metabolites	Valerate	−0.067	0.077	0.413	6.58	0.583
serum metabolites	X-12188	−0.022	0.033	0.521	17.6	0.485
serum metabolites	X-12231	−0.038	0.039	0.35	10.4	0.498
serum metabolites	10-heptadecenoate (17:1n7)	0.202	0.19	0.347	5.53	0.237
serum metabolites	Gamma-glutamylisoleucine	−0.041	0.03	0.192	23.6	0.425
serum metabolites	X-13183–stearamide	−0.155	0.058	0.029	2.63	0.956
serum metabolites	2-oleoylglycerophosphocholine	−0.027	0.053	0.623	12.0	0.682
serum metabolites	X-14304–leucylalanine	0.062	0.053	0.259	9.43	0.926
serum metabolites	X-14632	−0.061	0.027	0.014	0.04	0.710
serum metabolites	Phenylalanine	−0.046	0.033	0.167	32.3	0.453
serum metabolites	Total cholesterol in very large HDL	0.002	0.027	0.942	22.6	0.654
serum metabolites	Cholesterol esters in very large HDL	−0.007	0.029	0.822	21.3	0.673
serum metabolites	Free cholesterol in very large HDL	−0.005	0.034	0.89	10.5	0.981
serum metabolites	Total lipids in very large HDL	0.032	0.047	0.51	5.46	0.964

**TABLE 4:** Sensitivity analyses of the causal effect of immunophenotypes on myocarditis.

Genomics	Expoure	Test for directional horizontal pleiotropy			Cochran’s Q test	
		Egger intercept	SE	*P*-Value	Q	*P*-Value
immunophenotypes	HLA DR on monocyte	0.261	0.193	0.404	0.587	0.443
immunophenotypes	CD39^+^ secreting CD4 regulatory T cell	0.007	0.135	0.959	4.98	0.418
immunophenotypes	CD39^+^ secreting CD4 regulatory T cell	0.007	0.138	0.959	5.00	0.415
immunophenotypes	CD39 on CD39^+^ activated CD4 regulatory T cell	−0.041	0.131	0.777	2.35	0.503
immunophenotypes	CD39^+^ CD4^+^ T cell	−0.021	0.108	0.860	2.35	0.503
immunophenotypes	CD39^+^ CD4^+^ T cell Absolute Count	−0.041	0.101	0.709	3.48	0.481
immunophenotypes	CD33dim HLA DR+ CD11b-	−0.1	0.192	0.694	0.892	0.345
immunophenotypes	CD33dim HLA DR + CD11b	−0.1	0.192	0.694	0.892	0.345
immunophenotypes	CD25 on naive-mature B cell	−0.033	0.101	0.766	2.23	0.527

## 4 Discussion

As far as we know, this is the first study based on a publicly available database to explore the causal relationship between 211 gut microbiotas, 486 serum metabolites, 731 immunophenotypes and myocarditis.

Recent studies have proposed a link between gut microbes and heart health, the so-called “gut-heart axis” (Liu et al., 2018; Li et al., 2024). Among this MR analysis, a variety of gut microbiotas have been shown to be strongly associated with the inflammatory response. Clostridia, Ruminococcaceae and Bilophila may be negatively associated with the risk of developing myocarditis, while Lactobacillales and *Clostridium difficile* may be positively associated with the risk of developing myocarditis. It has been reported that Clostridia, a group of microorganisms known to influence intestinal barrier function and immunomodulation, are involved in anti-inflammatory processes through the production of short-chain fatty acids (e.g., butyric acid) (Stoeva et al., 2021; Chen et al., 2020a; Weng et al., 2015). Certain members of the Ruminococcaceae are also thought to be beneficial for the maintenance of intestinal health and immune homeostasis (Fu et al., 2020; Keshteli et al., 2022). Interestingly, while Lactobacillales have mostly served to reduce the inflammatory response in previous studies of a wide range of diseases, in the present study, Lactobacillales increased the risk of myocarditis (Li et al., 2023; Wang et al., 2020). It has been reported that *Lactobacillus* can intervene in excessive inflammatory responses throughout the body by altering the number, recruitment, and differentiation of immunophenotypes, as well as affecting the release and degradation of inflammatory factors by binding to specific recognition receptors (Zhang et al., 2023). Therefore, we hypothesize that although *Lactobacillus* has a modulatory effect on the immune system, however, the same studies have reported that in some cases it may also overstimulate the immune response, leading to immune-mediated inflammation, including cardiac effects (Ashraf and Shah, 2014; Torres-Chavez et al., 2023). *Clostridium*, a Gram-positive anaerobic bacterium, has been reported to secrete toxins that disrupt the epithelial cytoskeleton and tight junctions, increase epithelial permeability, and promote the production of inflammatory cytokines, thus playing a role in disease pathogenesis (Pruitt and Lacy, 2012; Pietrangeli et al., 2021). These microorganisms participate in the host’s inflammatory response and immune regulation through different mechanisms, showing the complex impact of gut microbial diversity on health. The above explanations are only hypothetical and the actual biological mechanisms are likely to be more complex and need to be validated by specialized studies.

The presence of several serum metabolites was found to be potentially causally related to myocarditis. Four types of “Free cholesterol in very large HDL”, “Total lipids in very large HDL”, “Total cholesterol in very large HDL” and “Cholesterol esters in very large HDL serum metabolites may be positively associated with the risk of developing myocarditis. We hypothesize that they may be primarily related to abnormal cholesterol metabolism, inflammatory response, and genetic factors. All four serum metabolites are used to assess lipid metabolism and the composition of lipid particles, and when these markers are elevated, they may lead to cholesterol deposition in cardiac tissues, triggering an inflammatory response and thus increasing the risk of myocarditis. At the same time, existing studies have reported that elevated lipid levels may lead to a systemic inflammatory response, and thus may similarly increase the risk of developing myocarditis (Khadge et al., 2018; Choy and Sattar, 2009). In addition, it can be speculated from a genetic perspective that these lipid parameters may be associated with specific metabolic pathways or pathogenesis of myocarditis. The study also found that 2-hydroxybutyrate is strongly associated with the risk of developing myocarditis. 2-hydroxybutyrate is a metabolite involved in fatty acid metabolism and ketone body metabolism, and is commonly associated with energy production processes. It has now been found that *Clostridium* has the ability to produce 2-hydroxybutyrate (Sharma et al., 2021; Di Marino et al., 2018; Castro et al., 2023). Coincidentally, in the present MR analysis of the gut microbiota, it was also found that *Clostridium* may have a potential causal relationship with myocarditis, but the complexity of the link still needs to be further explored (Wan et al., 2022).

This study also found that three types of immunophenotypes, CD39^+^ secreting CD4 regulatory T cell, CD39^+^ CD4 regulatory T cell, and CD39 on CD39^+^ activated CD4 regulatory T cell, all belong to the T cell category, but in fact perform different functions. For example, the CD39^+^ secreting CD4 regulatory T cell has the ability to suppress the immune response by secreting regulatory cytokines (e.g., IL-10 and TGF-β) to inhibit the activities of other immunophenotypes, whereas the CD39^+^ CD4 regulatory T cell regulates the immune response through the activity of the enzyme CD39, which converts excess ATP and ADP to AMP, thereby reducing the role of these excitatory molecules in the immune response. Based on previous studies, CD4 regulatory T cells were found to be a subpopulation of immunophenotypes, similar in function to CD39. It has been reported that CD4 regulatory T cells can secrete regulatory cytokines (IL-10 and TGF-β) to inhibit the activities of other immunophenotypes, and thus we speculate that CD4 regulatory T cells play an important role in myocarditis (Chen et al., 2003; Jutel et al., 2003; Wan and Flavell, 2008). Interestingly, both CD4 regulatory T cells and CD39 may be positively correlated with the risk of developing myocarditis, and it can be hypothesized that overexpression or aberrant activation of CD39 and CD4 regulatory T cells leads to a weakening of their inhibitory effects, causing abnormalities in the immune system, which then exacerbates immune responses, including inflammatory responses, in cardiomyocytes. Absolute CD39^+^ CD4^+^ T cell count is the absolute number of CD39^+^ CD4^+^ T cells and is also associated with regulatory T cells (Tregs). This is consistent with the previous trend in this study, so it can be hypothesized that there may be a link between an increase in the absolute CD39^+^ CD4^+^ T cell count and the onset or progression of myocarditis. CD25 is the alpha subunit of the IL-2 receptor, which is mainly associated with T cell activation and immunomodulation. CD25 on naive-mature B cells refers to a subpopulation of B cells that are not yet activated and at the same time not uninvolved in antibody production or immune response. It has also been shown that an increase in CD25 on naive-mature B cells reduces damage in myocarditis, which is consistent with previous findings (Ono et al., 2006; Wei et al., 2006; Chen et al., 2020b; Cen et al., 2021).

This study also suffers from a number of shortcomings, including the following three. 1. Population limitations: This study is based primarily on genetic data from a European population, which may limit the generalizability of the results. 2. Data type limitations: Because this study used secondary data analysis and relied on aggregated data from publicly available databases, it lacked detailed information at the individual level, such as lifestyle, dietary habits, and other potential confounders, which may affect the accuracy of causal inferences. Meanwhile, there are still “unknown” in the gut microbiota and serum metabolite data, which need to be further identified. 3. Potential mechanisms are not clear: Although the study identified potential causal associations between the gut microbiota, serum metabolites, and immunophenotypes with myocarditis, the specific biological mechanisms have not been fully elucidated.

This study has some implications for future clinical applications. Firstly, longitudinal studies are needed to confirm identified causal relationships and explore underlying mechanisms. Studies should also focus on different populations to ensure that findings are applicable to different genetic backgrounds. Secondly, findings based on gut microbiotas, serum metabolites and immunophenotypes could attempt to develop individualized strategies for the prevention and treatment of myocarditis. For example, in patients at high risk for myocarditis, the risk of morbidity could be reduced by altering the composition of the gut microbiotas or supplementing with specific metabolites. In addition to this, future studies could explore how these biomarkers could be used to personalize prevention and treatment strategies.

## 5 Conclusion

The MR study initially revealed potential causal associations between the gut microbiota, serum metabolites, and immunophenotypes with myocarditis risk. Although limited by the diversity of data sources and the limitations of the study population, these findings provide important clues for a deeper understanding of the etiologic mechanisms of myocarditis and the development of precision medicine strategies. Future studies are needed to validate these results in a wider population and to explore interventions based on these biomarkers through clinical trials to facilitate the prevention, early diagnosis, and treatment of myocarditis.

## Data Availability

The original contributions presented in the study are included in the article/Supplementary Material, further inquiries can be directed to the corresponding authors.

## Supplementary material

Supplementary Table S1 The gut microbiota dataset was screened for criteria that resulted in 178 SNPs.

Supplementary Table S2 caption published in website.

Supplementary Table S3 The serum metabolites dataset was screened for criteria that resulted in 361 SNPs.

Supplementary Table S4 The immunophenotypes dataset was screened for criteria that resulted in 51 SNPs.
